# Endothelial-Derived APT1-Mediated Macrophage-Endothelial Cell Interactions Participate in the Development of Atherosclerosis by Regulating the Ras/MAPK Signaling Pathway

**DOI:** 10.3390/life12040551

**Published:** 2022-04-07

**Authors:** Xinghua Wang, Lijun Cheng, Huaying Fu, Calista Zhuo Yi Chan, Gary Tse, Tong Liu, Guangping Li

**Affiliations:** 1 Tianjin Key Laboratory of Ionic-Molecular Function of Cardiovascular Disease, Department of Cardiology, Tianjin Institute of Cardiology, The Second Hospital of Tianjin Medical University, Tianjin 300211, China; wxh2022@tmu.edu.cn (X.W.); ydey_clj@tmu.edu.cn (L.C.); fuhuaying@tmu.edu.cn (H.F.); garytse@tmu.edu.cn (G.T.); 2 Department of Medicine and Therapeutics, Chinese University of Hong Kong, Hong Kong, China; calista@link.cuhk.edu.hk; 3 Li Ka Shing Institute of Health Sciences, Faculty of Medicine, Chinese University of Hong Kong, Hong Kong, China; 4 Kent and Medway Medical School, Canterbury CT2 7FS, UK; 5 Laboratory of Cardiovascular Physiology, Cardiovascular Analytics Group, Hong Kong, China

**Keywords:** atherosclerosis, APT1, miR-138-5p, extracellular vesicles, Ras/MAPK signaling pathway

## Abstract

Acyl-protein thioesterase 1 (APT1) can affect H-Ras localization and function by promoting its depalmitoylation. However, relatively little attention has been paid to the effects of APT1 on H-Ras in the cardiovascular system. In this study, we revealed its roles in atherosclerosis development using oxidative low-density lipoprotein (ox-LDL)-induced endothelial dysfunction models and a Western diet-induced ApoE^−/−^ mouse model. The results showed that APT1 expression was up-regulated, while that of miR-138-5p (miR-138) was down-regulated (*p* < 0.05) in this model. In the meantime, APT1 and H-Ras were translocated from the cytoplasm to the plasma membrane. Bioinformatic analysis and double fluorescence identified miR-138 as the upstream regulator of APT1. APT1 knockdown regulated H-Ras localization and expression, which subsequently affected the MAPK signaling pathway and the expression of its downstream factors. Further research indicated that human umbilical vein endothelial cells (HUVECs)-derived biogenic nanoparticles (BiNPs), hBPs secretion, and RNA expression of hBP-loaded APT1 were increased (*p* < 0.05) in the ox-LDL induced endothelial dysfunction model. Meanwhile, the HUVECs-derived APT1 could further affect macrophage function through hBP transportation. Altogether, this study demonstrated that the miR-138-APT1 axis may be partially responsible for atherosclerosis development by regulating the H-Ras-MAPK signaling pathway and hBP transportation. The results also shed novel insight on the underlying mechanisms of, and identify potential diagnostic and therapeutic targets for, atherosclerotic cardiovascular diseases in the future.

## 1. Introduction

Acyl-protein thioesterase (APT1), known as LYPLA1, a 25 kDa acyl protein that has been described as a lysophospholipase [1,2], is also one of the most common thioesterases known to be able to depalmitoylate [3,4,5]. Although APT1 exhibits enzyme activities in both respects, its effect on lysophospholipids is significantly lower (~100×) than that on palmitoyl-protein [6,7]. Palmitoylation is the only reversible lipid modification, and this dynamic acylation cycle is a reaction-diffusion system, which governs the transient membrane association of peripheral membrane proteins, including the trimeric G proteins, Wnt, and Ras proteins, thereby regulating their trafficking and steady-state localization [8,9,10]. APT1 is a major factor in protein depalmitoylation, and it is involved in most path physiological processes, such as differentiation, immune response, proliferation, and migration [11,12,13,14]. The depalmitoylation activity of APT1 was itself dependent on membrane localization [15]. One of the earliest identified targets of APT1 is H-Ras, a protein implicated in numerous diseases, including cancer and cardiovascular disease. RAS proteins (K-Ras4A, K-Ras4B, N-Ras, and H-Ras) consist of small GTPases (guanosine triphosphatases) that act as molecular switches, transducing extracellular signals from activated receptors at the cell surface through various signaling pathways to the nucleus, thus regulating cell proliferation, survival, and differentiation [16]. Mutations in Ras genes are found in 25% of human cancers; however, H-Ras is the least frequently mutated Ras isoform in human cancers (3%), compare with K-Ras (85%), and N-Ras (11%) [17]. Meanwhile, there were more palmification sites (two) in H-Ras than in N-Ras and K-Ras4B (one), so we suggested that H-Ras altered membrane localization by depalmitoylation, significantly affecting its function and downstream signaling pathways, such as the MAPK signaling pathway.

MicroRNA (miRNA) (~22 nt) is the one of the most fundamental regulatory non-coding RNAs. It is involved in pathology, physiology, and development, and it plays an important role in the development of cardiovascular disease [18,19]. The existing research on miR-138, a key factor in this process as an upstream factor of APT1, focuses on the field of tumors, since it can act as a tumor suppressor gene by inhibiting cell proliferation, promoting apoptosis, and inhibiting metastasis and invasion [20]. Sarah U [21] and Wu [22] et al. demonstrated that miR-138 was involved in the normal development of the heart, while Li [23] and He [24] et al. found that miR-138 can protect hypoxia-induced cardiomyocyte injury and pulmonary artery smooth muscle cells by inhibiting cell apoptosis.

Endothelial cells and macrophages, the main players during the development of atherosclerosis, interact and synergistically affect the occurrence and development of atherosclerosis [25,26]. The extracellular vesicles (EV) are biogenic nanoparticles (BiNPs) that are prominent mediators for the communication between these two different types of cells via the transportation of nucleic acids, proteins, carbohydrates and lipids [27,28], making endothelial and macrophage intercellular signaling possible. In this study, we revealed that APT1 was released with BPs derived from endothelial cells, which is further transferred to macrophages to exert corresponding physiological functions. APT1 links the various cells and causes these cells to interact with each other to participate in the development of atherosclerosis. The findings of this study aim to shed light on the underlying mechanisms and to identify potential diagnostic and therapeutic targets for atherosclerosis.

## 2. Results

### 2.1. APT1 Is a Crucial Factor in Atherosclerosis

**Figure 1:**
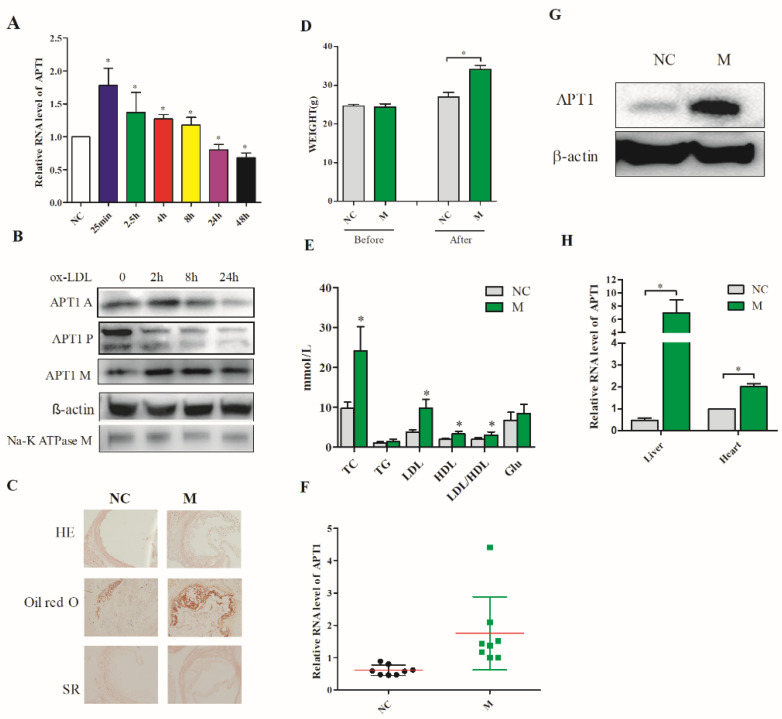
Expression of APT1 in atherosclerosis model. (**A**,**B**), APT1 expression level in human umbilical vein endothelial cells (HUVEC); (**C**), pathological staining of aorta; (**D**,**E**), blood lipids and mouse body weight results; (**F**,**G**), APT1 expression level in aorta; (**H**), The RNA expression level of APT1 in heart and liver tissue. A: whole cell; M: membrane; P: cytoplasm; HE: hematoxylin and eosin staining; SR: Sirius red staining; NC: control group; M: model group. The results are shown by mean ± standard deviation; the above experiments are repeated three times; * represents *p* < 0.05.

The in vitro atherosclerosis cell model was constructed using 50 μg/μL oxidative low-density lipoprotein (ox-LDL) in endothelial. APT1 expression levels were measured by qPCR and Western blotting. The results indicated APT1 expression levels were increased compared to the normal group (*p* < 0.05) in vitro; meanwhile, APT1 localization was changed to the plasma membrane in the atherosclerosis group (Figure 1B). Subsequently, the animal model was established by feeding the ApoE^−/−^ mice with a Western diet (Figure 1C–E). APT1 expression levels were increased in multiple organs (aorta, heart, and liver) in the atherosclerosis group (M group) compared to the control group (NC) (Figure 1F,G) in vivo.

### 2.2. miR-138 Is an Upstream Factor of APT1

**Figure 2:**
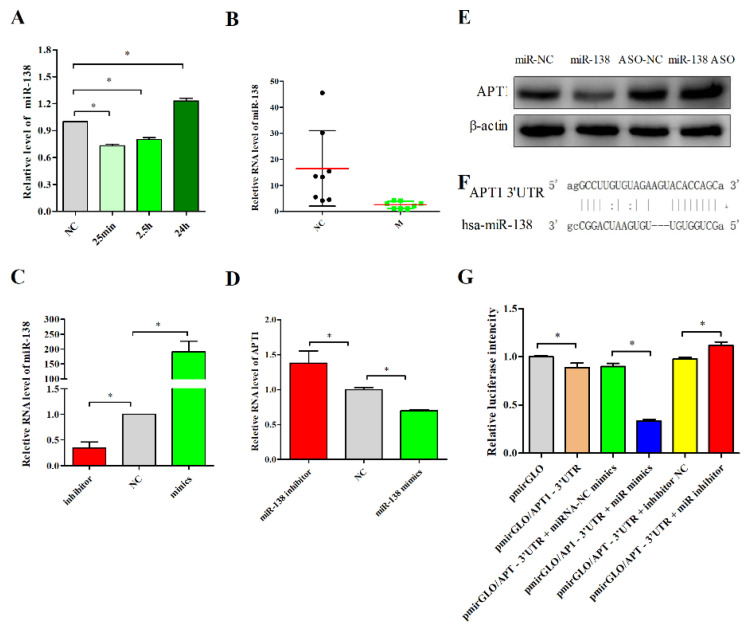
miR-138 is the upstream acting factor of APT1. (**A**), miR-138 expression level in HUVEC; (**B**), miR-138 expression level in aorta; (**C**), The expression level of miR-138; (**D**), the RNA expression level of APT1; (**E**), the protein expression level of APT1; (**F**), target relationship between miR-138 and APT1; (**G**), dual fluorescence reporter vector assay for APT1 and miR-138 direct target relationship. The results are shown by mean ± standard deviation; the above experiments are repeated three times; * represents *p* < 0.05.

The bioinformatics method was used to search for the upstream action factor of APT1 in the DIANA-microT [29], TargetScanHuman [30], and miRDB databases [31], all of which yielded miR-138, miR-23b-3p, miR-23c, miR-23a-3p, and miR-135b-5p (Table S1) as the predicted result. Among these six RNAs, miRNA-138 is associated with myocardial development and cardiomyocyte injury, as well as lipid metabolism [21,22,23,24,32]. Thus, we detected the expression level of miR-138 in the atherosclerosis model, consequently discovering the expression level of miR-138 to be inversely related to that of APT1 (Figure 2A,B). To further validate the relationship between miR-138 and APT1 and their roles in atherosclerosis, we detected the expression of APT1 by the overexpression and inhibition of miR-138 expression and found that APT1 is inversely associated with miR-138 expression; overexpression of miR-138 can inhibit APT1, and vice versa (*p* < 0.05) (Figure 2D,E).

Subsequently, to further verify the direct targeting relationship between miR-138 and APT1, we constructed a plasmid containing the binding site of miR-138 (Figure 2F) and performed a luciferase activity assay. The results revealed that the overexpression of miR-138 could significantly inhibit the fluorescence intensity. On the contrary, the inhibition of miR-138 expression enhanced the fluorescence intensity, which confirmed the direct targeting relationship between miR-138 and APT1 (Figure 2G).

**Figure 3:**
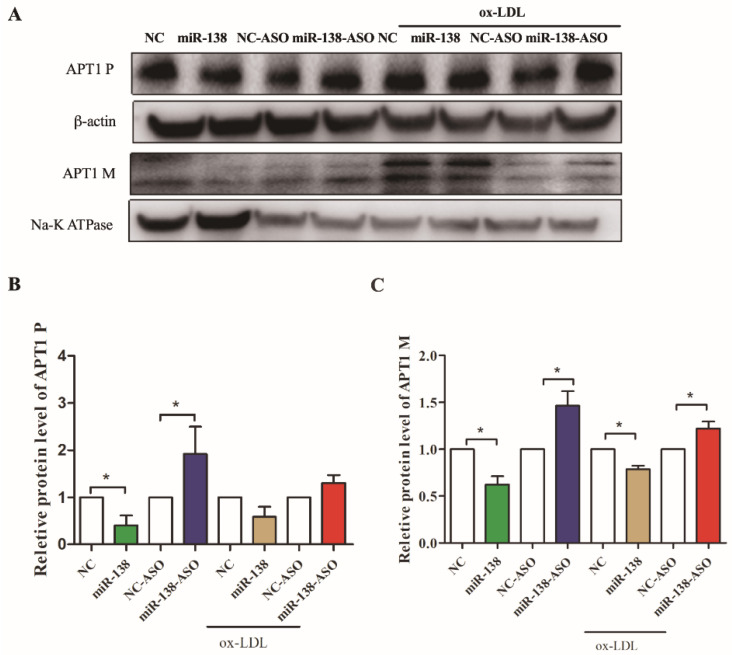
Expression levels of different cell sublocalizations and of APT1 after overexpression or inhibition of expression of miR-138. (**A**), expression level of APT1 after overexpression or inhibition of expression of miR-138; (**B**), quantitative analysis of Figure (**A**) for cytoplasmic expression of APT1; (**C**), quantitative analysis of Figure (**A**) for membrane expression of APT1. Results are shown by mean ± standard deviation; the above experiments were repeated three times; * represents *p* < 0.05.

Finally, the study aimed to reveal whether such a link was present in different cellular localizations in a cellular model of atherosclerosis. The results showed that in the untreated human umbilical vein endothelial cells (HUVEC), APT1 expression was decreased under the miR-138 overexpression condition in both the cytoplasm and the membrane (*p* < 0.05) (Figure 3A–C), whereas increased miR-138 did not visibly inhibit APT1 in the plasma after ox-LDL stimulated HUVEC (*p* > 0.05) (Figure 3A–C). As such, we concluded that the inhibition of miR-138 expression could increase APT1 expression in both the cell membrane and the cytoplasm under normal conditions and ox-LDL induced cell membrane, while APT1 overexpression in the ox-LDL group prevented the inhibitory effect of miR-138 on APT1, which reaffirmed the increased expression of APT1 in the atherosclerosis models.

### 2.3. H-Ras Is a Downstream Factor of APT1

APT1, a major protein for depalmification modification, plays an important role in the transport, localization, and function of H-Ras. Dekker et al. found that the application of APT1 inhibitors allowed the palmification of H-Ras to be localized on the membrane, altering the correct localization and distribution of H-Ras in cells and in its downstream signaling pathways [3]. This reverses the change in tumor phenotype caused by the overexpression of H-Ras [3,33]. Therefore, APT1 is closely related to the activity and function of H-Ras. However, whether the alteration of the Ras signaling pathway activity in the cardiovascular system is related to its palmification modification has been rarely reported.

**Figure 4:**
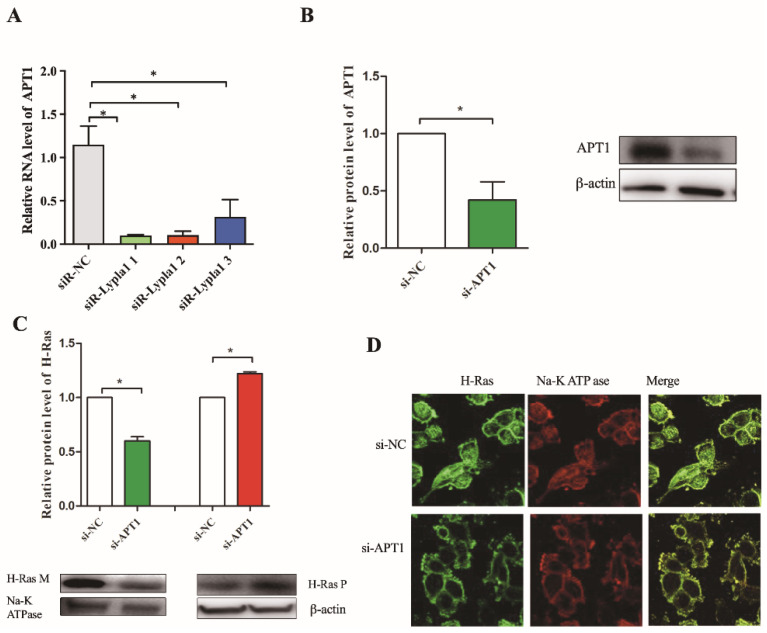
Inhibition of APT1 expression affects cell membrane localization of H-Ras. (**A**), Different APT1 siRNAs, labeled 1, 2, and 3, respectively, after transfecting with HUVEC; the expression of APT1 RNA was detected using real-time PCR. We found that siRNA 1 had the greatest effect on the expression of APT1 RNA. Subsequently, siRNA 1 was used in the experiment; (**B**), HUVEC was transfected with siRNA 1, and Western blotting detected APT1 protein expression; (**C**), To detect the expression level in the cell membrane and cytoplasm of H-Ras after the inhibition of APT1; (**D**), shows the expression of H-Ras by fluorescence confocal experiments after inhibition of expression of APT1. Na-K ATPase: the internal reference protein of the cell membrane; M: the membrane; P: the cytoplasm. The results are shown by mean ± standard deviation; the above experiment was repeated three times; * represents *p* < 0.05.

To verify the effect of APT1 on the expression and localization of H-Ras, loss-of-function mutations were introduced to the APT1 gene. The results showed that decreased APT1 did not obviously increase the expression of H-Ras in the cell cytoplasm, but decreased the expression of H-Ras in the cell membrane (Figure 4C,D). Because APT1 can return the intracellularly mislocated H-Ras back to the Golgi apparatus, which will then be reused after entering a new palmitoyl cycle, decreased APT1 expression will reduce the re-palmitoylation of H-Ras and its localization to the cell membrane, resulting in a higher expression of H-Ras in the cytosol and a lower expression of H-Ras in the membrane. To determine whether the activity of the downstream-related signaling pathway increased, we conducted the subsequent study by inhibiting the expression of APT1.

### 2.4. APT1 Can Affect the MAPK Signaling Pathway and Related Factor Expression

**Figure 5:**
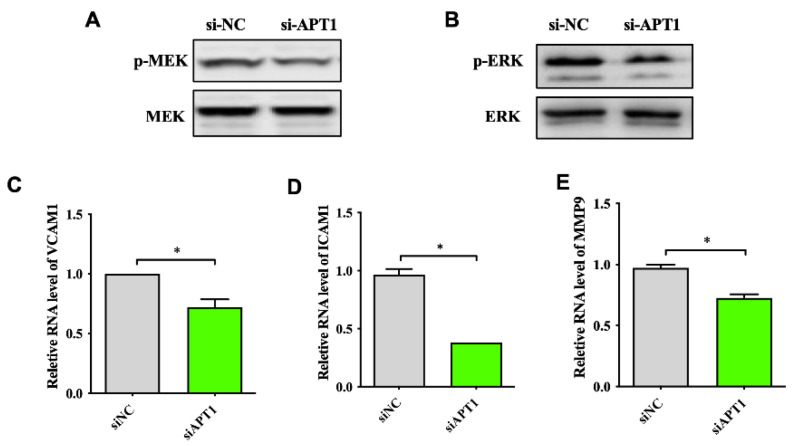
Effect of inhibition of expression of APT1 on downstream signaling pathways and inflammatory factors. (**A**), p-MEK protein expression level; (**B**), p-ERK protein expression level; (**C**–**E**), three inflammatory factor RNA expression levels. The results are shown by mean ± standard deviation; the above experiments were repeated three times; * represents *p* < 0.05.

Since atherosclerosis is an inflammatory reaction, factors such as matrix metalloproteinases (MMPs), the adhesion proteins intercellular adhesion molecule 1 (ICAM-1), and the vascular cell adhesion molecule 1 (VCAM-1) are associated with atherosclerotic plaque formation and thus, the instability of vulnerable plaques. In vitro experiments have shown that the activation of extracellular signal-regulated protein kinase ERK1/2 can promote the secretion of inflammatory factor MMP-9. Therefore, the mitogen-activated protein kinase (MEK)/ERK pathway can promote the development of atherosclerosis by regulating the expression of MMP-9 [34]. In addition, the activation of ERK1/2 can also increase the expression of adhesion factors VCAM-1 and ICAM-1 by increasing the expression of transcription factors such as nuclear factor kappa B (NF-κB) in the nucleus. The Ras gene is an upstream regulator of the MEK/ERK pathway, so we hypothesized that APT1 can influence the expression of related inflammatory factors through the Ras-MAPK pathway and ultimately participate in the development of atherosclerosis. To validate the effect of APT1 on the H-Ras downstream signaling pathways, we measured the expression of p-ERK (Figure 5A,B) and its downstream atherosclerosis-related factors, such as MMP-9, VCAM-1, and ICAM-1 (Figure 5C–E). We found that the expression of p-MRK, p-ERK, and downstream inflammatory factors was decreased after the inhibition of APT1 (*p* < 0.05).

**Figure 6:**
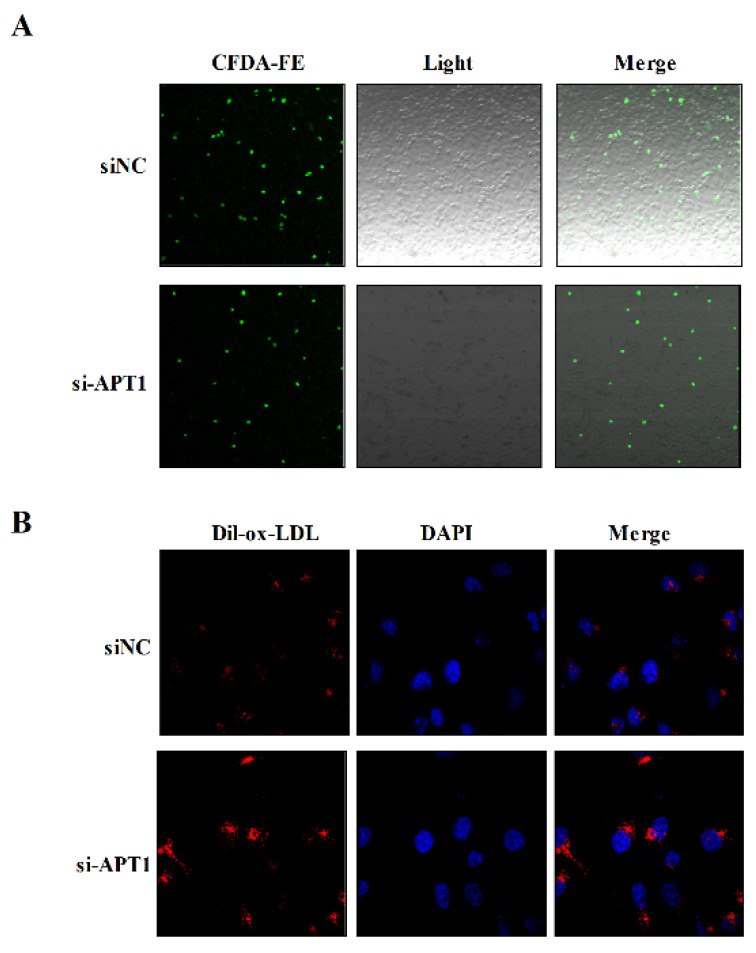
Effects of inhibition of expression of APT1 on atherosclerosis; (**A**), fluorescence microscopy method to detect the adhesion ability of macrophages; (**B**), fluorescence microscopy method to detect lipid phagocytosis ability of HUVEC cells.

To further illustrate the role of APT1 in atherosclerosis development, relevant phenotypic studies were carried out. The results found that the ability of THP-1 to adhere to the endothelial cells was inhibited in the APT1 siRNA group (Figure 6A), but the phagocytic capacity of ox-LDL was increased in the HUVEC cells (Figure 6B).

### 2.5. Effect of Endothelium Derived APT1 on Macrophages

Satou [35] et al. revealed that APT1 was released at high levels from RAW264.7 cells into the culture medium after stimulation with lipopolysaccharide (LPS). We therefore investigated whether APT1 could be transferred into a macrophage by HUVECs-derived biogenic nanoparticles (BiNPs), further affecting the function of the macrophages.

**Figure 7:**
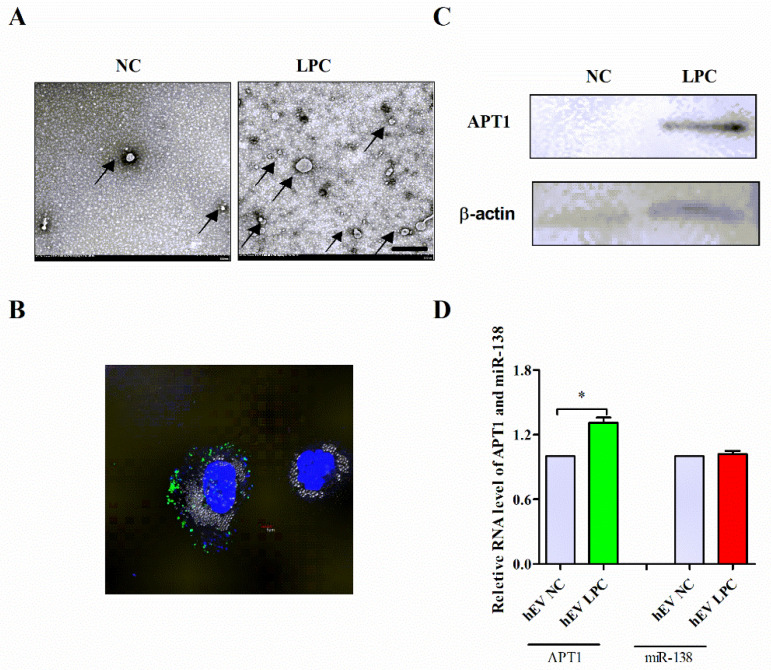
Effect of APT1 in endothelium-derived hBPs on macrophages. (**A**), The number and morphology of BPs under electron microscopy in normal group and LPC-stimulation group, where the scale bars were 500 nm; (**B**), confocal observation fluorescently labeled BPs are phagocytosed by macrophages; (**C**), expression of APT1 protein in BPs after LPC stimulation; (**D**), the RNA expression of APT1 and miR-138 in BPs after LPC stimulation. The results were all shown by mean ± standard deviation; the above experiments were repeated three times; * represents *p* < 0.05.

The HUVECs were divided into two groups—the normal control group (NC) and the lysophosphatidylcholines (LPC) stimulation group (LPC)—and the hBPs were isolated by low-temperature ultracentrifugation. The microscopic observation of its morphological characteristics revealed that the endothelial-derived BPs have a circular or elliptical membranous vesicular structure, with a diameter between 100 and 1000 nm (Figure 7A). Subsequently, the hBPs were fluorescently labeled and co-cultured with the THP-1 macrophages. A large number of hBPs were uptaken after 3 h of co-culture, as shown in Figure 7B. Then, the expression of miR-138 and APT1 in hBPs was measured by qPCR and Western blotting. The study results revealed that APT1 expression was increased (*p* < 0.05) (Figure 7C,D) compared with the NC group, while there was no significant difference in miR-138 expression (*p* > 0.05) (Figure 7D) even after optimization conditions.

## 3. Discussion

Noncoding RNAs (ncRNAs), which were once thought to be useless products of the genome, have garnered increasing attention in recent years due to their significant physiological and pathological functions. Many previous studies have examined their functions in the context of cancer, chronic inflammatory processes, and degenerative conditions [36,37,38]. However, they are increasingly recognized to be important in the pathogenesis of cardiovascular diseases [39,40,41]. In this study, we measured the expression levels of APT1 and miR-138 in the ox-LDL-induced HUVECs model and further verified their direct targeting relationship by dual fluorescence reporter assays.

Subsequently, the APT1 downstream factor H-Ras was identified. Studies in the cardiovascular field have shown that elevated Ras signaling pathway activity is closely related to the development of atherosclerosis [42]. In a mouse model of atherosclerosis, alpha-lipoic acid (ALA) can attenuate the progression of atherosclerosis by inhibiting the proliferation of vascular smooth muscle cells through the targeting of the Ras-MEK1/2-ERK1/2 pathway [43]. Numerous studies have shown that APT1 is the crucial palmitoyl thioesterase of the H-Ras palmitoyl cycle. In this study, we elaborate that the APT1 can become involved in the Ras-MAPK signal pathways by affecting H-Ras localization. APT1 suppression resulted in decreased H-Ras expression in the cell membrane, along with attenuated MEK/ERK activation and downstream targeting (MMP-9, VCAM-1, and ICAM-1).

During the development of atherosclerosis, endothelial cells and macrophages interact and synergistically affect the occurrence and development of atherosclerosis [25,26]. To further verify the effect of APT1 on atherosclerosis, we co-cultured THP-1 macrophages with HUVECs to detect the effect of APT1 on the macrophage adhesion and lipid phagocytosis of endothelial cells. BPs as mediators for the communication between different cells and tissues are involved in the pathological processes of various diseases such as atherosclerosis, diabetes, inflammation, nervous system disorders, and malignant tumors [44,45]. A study by Li et al. [46] found that the use of cigarette extracts to stimulate monocytes can activate the Ras-MAPK signaling pathway to produce BPs. This indicates that the Ras-MAPK signaling pathway plays an important role in the production of BPs. Because the MAPK signaling pathway was activated in the atherosclerosis model, we finally focused on determining whether APT1 can be transferred to the THP-1 macrophage by hBPs and how it affects macrophage function in atherosclerosis. Our study showed that the hBPs can not only be rapidly swallowed by THP-1 macrophages, but that their number also increased significantly, accompanied by the enhanced RNA and protein expression of hBPs-derived APT1 compared with the control group, while there was no statistical difference in the expression of miR-138. We hypothesized that hBPs-derived APT1 may enter macrophage-associated signaling pathways (such as Ras-MAPK-MMP-9) and further affect the development of atherosclerosis.

It is known that atherosclerosis is a complex, multi-factor, multi-tissue systemic disease. In this study, we found that the miR-138-APT1 axis may be partially responsible for atherosclerosis development by regulating the H-Ras-MAPK signaling pathway and hBPs transportation. APT1 crosses cell and tissue barriers by BPs transportation, enabling these cells to interact with each other to participate in the development of atherosclerosis.

## 4. Materials and Methods

### 4.1. Cell Culture, Plasmids, and Adenoviral Vectors

The human umbilical vein endothelial cells (HUVECs) CRL-1730 and THP1 were obtained from the American Type Culture Collection and maintained in DMEM-F12 (Gibco, Waltham, MA, USA) and 1640 (Gibco, Waltham, MA, USA) supplemented with 10% fetal bovine serum (FBS) (Gibco, Waltham, MA, USA), 100 μg/mL streptomycin, and 100 IU/mL penicillin and maintained at 37 °C in a humidified atmosphere with 5% CO_2_. The atherosclerotic model was stimulated with HUVEC with 50 μg/μL of ox-LDL (Yiyuan Biotechnologies, Guangzhou, China) at different time points.

### 4.2. Animal Experiment

A total of 20 male ApoE^−/−^ mice with the C57BL6 background, aged between 8 to 10 weeks, were purchased from Beijing Weitong Lihua Experimental Animal Technology Co., Ltd. The Laboratory Animal License No.is SCXK (Beijing) 2016-0006. Before the experiment, 20 mice were randomly divided into two groups and weighed. The two groups were: the control group (group C, n = 8), and the high-fat/high-cholesterol feeding group (group M, n = 12). Our animal experimental protocol was approved by the Experimental Animal Administration Committee of Tianjin Medical University and Tianjin Municipal Commission for Experimental Animal Control.

After 15 weeks of feeding, the animals were sacrificed, and specimens were collected as needed. Enzymatic assays were used to measure the plasma levels of total cholesterol (TC), triglycerides (TG), low-density lipoprotein cholesterol (LDL), high-density lipoprotein cholesterol (HDL), and glucose (Glu) (Biosino Biotech, Beijing, China) in blood samples from the two groups of mice. Aorta samples for RT-PCR were snap-frozen in liquid nitrogen immediately after collection, then stored at −80 °C. Samples of aortic roots were embedded in Tissue-Tec OCT and snap-frozen for cryosectioning. Embedded hearts were sectioned into 6 μm thick slices and then placed in a −80 °C refrigerator for subsequent experiments.

### 4.3. Histology, Oil Red O, and Picro Sirius Red Staining

The lesion area was determined using hematoxylin and eosin (H&E) stained outflow tract sections, performed as previously described. Collagen formation was detected by picro-sirius red staining (Solarbio, Beijing, China) following the manufacturer’s instruction; cross-sections of aortic roots were stained with Oil red O to assess lipid accumulation. Images were captured by microscopy.

### 4.4. RNA Extraction and Quantitative Real-Time RT-PCR (qRT-PCR) Analysis

Total RNA was extracted from cells, frozen tissue samples, or BPs using TRIzol reagent (Life Technologies, Grand Island, NY, USA) according to the manufacturer’s instructions. For miRNA-138 detection, 1 μg RNA was reverse transcribed to cDNA using M-MLV reverse transcriptase (Promega, Madison, WI, USA) with miR-138-specific primer. To detect the mRNAs expression level, 3 μg RNA was reverse transcribed to cDNA using oligo (dT) primers and M-MLV reverse transcriptase (Promega, Madison, WI, USA). The qRT-PCR analysis was performed in triplicate with the SYBR Green Master Mix (Roche, Pleasanton, CA, USA), according to the manufacturer’s instructions, to determine the expression levels of RNA. All primers are listed in Table S2 U6 RNA was used to normalize the expression of miRNA, and β-Actin or S26 were used to normalize expression of mRNAs.

### 4.5. Plasmids and Adenoviral Vectors

Wild-type APT1and lacZ were cloned into GV314 to generate adenoviruses expressing APT1 (Ad-Chaer) and lacZ (Ad-lacZ). APT1-3′UTR was inserted into the Pmir GLO vector using PmeI and Xbal for in vitro transcription. Lipofectamine 3000 (Life Technologies, Grand Island, NY, USA) was used to transfect plasmids or oligonucleotide into the HUVEC cells, according to the manufacturer’s instructions. Negative control siRNA (si-NC; GenePharma, Hangzhou, China), APT1 siRNA (si-APT1; designed and synthesized by GenePharma, Hangzhou, China), Negative control miRNA (MiR-138 NC; GenePharma, Hangzhou, China), MiR-138 mimics (GenePharma, Hangzhou, China), Negative control miRNA inhibitor (miR-138 inhibitor NC; GenePharma, Hangzhou, China), and miR-138 inhibitor (GenePharma, Hangzhou, China) were transfected into the HUVEC cells using Lipofectamine 3000 (Life Technologies, Grand Island, NY, USA). The HUVEC cells were infected with Ad-APT1 (107 PFU/mL) for overexpression with the same amount of Ad-lacZ as a control. Finally, ox-LDL (50 μg/μL) was used to induce the atherosclerotic model in HUVEC, and the macrophage model was stimulated with THP1 with PMA (final concentration 5 μg/L).

### 4.6. Western Blot Analysis

The cells and BPs were washed twice with ice cold PBS, and then harvested in RIPA lysis buffer with 1 mM phenylmethylsulfonyl fluoride. An equal amount of protein was loaded onto an 8–16% Bis-Tris gel (Life Technologies, NY, USA), separated by electrophoresis, transferred to a PVDF membrane (Merck Millipore, Billerica, MA, USA), and incubated with the specific primary antibodies anti-APT1 (Abcam, Cambridge, MA, USA), anti-H-Ras (Abcam, MA, USA), anti-Na-K GTPase (Santa Cruse, TX, USA), and anti-β-actin (Abcam, Cambridge, MA, USA) overnight at 4 °C. The membranes were then washed and subsequently incubated with the secondary antibody conjugated to horseradish peroxidase (HRP). Protein signals were visualized using HRP-conjugated secondary antibodies and enhanced chemiluminescence (ECL) Western blotting detection reagents (Thermo Fisher Scientific, Waltham, MA, USA). The resulting Western blot bands were quantified using the Image J software.

### 4.7. Luciferase Activity Assay

The HUVECs, cultured to sub-confluence in 6-well plates, were transfected with pmirGLO/APT1-3′UTR or pmirGLO, together with miR-138 mimics/NC or ASO-miR-138/ASO-NC using Lipofectamine 3000. After 48 h of transfection, cells were harvested to measure luciferase activities using the Dual-Luciferase Reporter assay system (Progema, Madison, WI, USA), according to the manufacturer’s protocol.

### 4.8. Fluorescence Microscopy Analysis of Monocyte Adhesion and Dil-Ox-LDL in HUVECs

Monocyte adhesion was analyzed as described previously, but with modifications [47]. Briefly, HUVECs were cultured in 24-well plates, transfected with si-NC and si-APT1, for 24 h. THP1 cells were labeled with CFDA, SE (US Everbright, Suzhou, China), according to the manufacturer’s protocol., then added to plates at 0.5 × 10^6^ cells per well. After incubation for 30 min at 37 °C, non-adherent cells were removed by washing three times with PBS. Following washing, three replicate wells for each group were incubated with 4% paraformaldehyde for 10 min, and the cells were washed three times with PBS. The image of monocyte adhesion was collected under a fluorescence microscope.

HUVECs (3 × 105 cells/well) were seeded into 24-well plates in triplicate 18 h prior to transfection with si-NC and si-APT1 for 24 h. Ox-LDL (50 µg/mL) was added to the cells and the cells were incubated for an additional 24 h. To visualize and assess ox-LDL uptake, the HUVECs were incubated with Dil-ox-LDL for 4 h at 37 °C. Following incubation, three replicate wells for each group were incubated with 4% paraformaldehyde for 10 min, and the cells were washed three times with PBS. The average fluorescence intensity of each group was observed by fluorescence microscope.

### 4.9. BP Isolation and BP-Depleted FBS Preparations

The BPs were isolated from HUVSCs with and without LPC stimulation. HUVECs supernatant were centrifuged twice at 1500× *g* for 10 min to eliminate cell debris. Next, the supernatant was ultracentrifuged at 100,000× *g* for 60 min at 4 °C (Rotor: SW 41Ti; Beckman Coulter, Brea, CA, USA). After carefully removing the supernatant, the pellets were suspended in 50 μL of sterile PBS and either immediately used or stored at −80 °C until further use.

The BP-depleted FBS was prepared following the same centrifugation procedure as above, and the supernatant was collected for further BP-associated experiments.

### 4.10. Transmission Electron Microscopy (TEM)

The hBPs were fixed with 4% paraformaldehyde and 4% glutaraldehyde in 0.1 M phosphate buffer (pH 7.4) and kept at 4 °C until analysis by TEM. A drop of each BP sample was placed on a carbon-coated copper grid and immersed in 2% phosphotungstic acid solution (pH 7.0) for 30 s. The stained BPs samples were analyzed with a transmission electron microscope (Hitachi H-7600, Hitachi Ltd., Tokyo, Japan).

### 4.11. BP Labeling and Cell Uptake Assay

The resuspended BPs were labeled with PKH67 (Sigma-Aldrich, St. Louis, MO, USA), according to the manufacturer’s protocol, with some modifications. Briefly, the BPs and PKH67 were separately diluted in 100 µL diluent C. The BPs were mixed with the staining solution and incubated at room temperature for 3 min. An equal volume of 10% BSA was used to neutralize excessive dye from the labeled BPs. The samples were ultracentrifuged to remove free PKH67. The labeled BPs were resuspended and incubated with THP1 for 3 h and washed with PBS. The nuclei were stained by DAPI (Beijing Solarbio Life Science, Beijing, China) for five minutes. A confocal microscope (Thermo Fisher Scientific, Waltham, MA, USA) was used to image the cells.

### 4.12. Statistics

The data are expressed as the mean ± standard deviation from at least three separate experiments. Comparisons between multiple groups were analyzed with one-way ANOVA, followed by a Student’s *t*-test to calculate the *p*-value between two groups. A two-tailed result of *p* < 0.05 was considered as statistically significant.

## Data Availability

Data is contained within the article or is available from the authors upon reasonable request.

## Supplementary Materials

The following supporting information can be downloaded at: https://www.mdpi.com/article/10.3390/life12040551/s1, Table S1: Bioinformatics predicts that candidate miRNAs that can target APT1; Table S2: Oligonucleotides used in this work.

## Sample Availability

Samples of the compounds can be requested and obtained from the indicated companies.
