# Supplementary material for: Endothelial-Derived APT1-Mediated Macrophage-Endothelial Cell Interactions Participate in the Development of Atherosclerosis by Regulating the Ras/MAPK Signaling Pathway
Source: Life (Basel). 2022 Apr 7;12(4):551. doi: 10.3390/life12040551 (PMC9026782; doi:10.3390/life12040551)
Supplement: Supplementary file 1 [file life-12-00551-s001.zip › Table S2.pdf]

Supplementary Material of Endothelial-Derived APT1-Mediated Macrophage–Endothelial Cell Interactions Participate in the Development of Atherosclerosis by Regulating the Ras/MAPK Signaling Pathway

Table S2. Oligonucleotides used in this work.

Name	Sequence (5′ → 3′)
**RT-PCR**	
Hsa/Mus-U6-RT	GTCGTATCCAGTGCAGGGTCCGAGGTATTCGCACTGGATACGACAAAA-TATGGAAC
Hsa/Mus-miR-138-RT	TCGTATCCAGTGCAGGGTCCGAGGTGCACTGGATACGACCGGCCTGA
Hsa/Mus-U6-forward	TGCGGGTGCTCGCTTCGGCAGC
Hsa/Mus-miR-138-forward	TGCGGAGCTGGTGTTGTGAATCA
Reverse (universal)	CCAGTGCAGGGTCCGAGGT
Hsa-Actin forward	CGTGACATTAAGGAGAAGCTG
Hsa-Actin reverse	CTAGAAGCATTTGCGGTGGAC
Mus-S26F-forward	TCATTCGGAACATTGTAGAAGCC
Mus-S26F-reverse	AGCTTGACATAGAGCTTGGGAA
Hsa-APT1-forward	CAGAAACTGGCAGGTGTCAC
Hsa-APT1-reverse	GGTCACATTGGCTGGATTCA
Mus-APT1-forward	CAGGCAGCAGAAACCGTAAA
Mus-APT1-reverse	CAGTGACACCAGCCAGTTTC
Hsa-VCAM1-forward	AAAAGCGGAGACAGGAGACA
Hsa-VCAM1-reverse	AAAAGCGGAGACAGGAGACA
Hsa-ICAM1-forward	TCTTCCTCGGCCTTCCCATA
Hsa-ICAM1-reverse	AGGTACCATGGCCCCAAATG
Hsa-MMP9-forward	CTCTGGAGGTTCGACGTGAA
Hsa-MMP9-reverse	TCAACTCACTCCGGGAACTC
**Transfection**	
Hsa-miR-138 mimics-sense	AGCUGGUGUUGUGAAUVAGGCCG
Hsa-miR-138 mimics-antisense	GCCUGAUUCACAACACCAGCUU
Hsa-miR-138 inhibitor	CGGCCUGAUUCACAACACCAGCU
Negative control Sense	UUCACCGAAAGUGACACGUTT
Negative control antisense	ACGAGACACGUUAGGAGCATT
MicroRNA inhibitor N.C	CAGUACUAUUGUGUCGUACUA
**Construction of plasmid**	
APT1 -3′UTR-Top	AAACTAGCGGCCGCTAGT CCTTGTGTAGAAGTACACCAGCAT

Name	Sequence (5′ → 3′)
	**RT-PCR**
APT1 -3′UTR-Bottom	CTAGATGCTGGTGTACTTCTACACAAGGACTAGCGGCCGCTAGTTT
APT Sense	TACCGGACTCAGATCTCGAGCGCCACCATGTGCGGCAA-TAACATGTCAAC
APT Antisense	GATCCCGGGCCCGCGGTACCGTATCAATTGGAGGTAGGAGTTTATC

Restriction sites are underlined.
